# Negative Afterimages From Flicker-Augmented Colors

**DOI:** 10.1177/2041669517699414

**Published:** 2017-03-31

**Authors:** Stuart Anstis

**Affiliations:** University of California, San Diego, La Jolla, CA, USA

**Keywords:** afterimage, color, flicker, contrast

## Abstract

A patch that alternates between two hues such as dark green and light blue looks greenish on a light gray surround and bluish on a dark gray surround (“flicker-augmented contrast”). Thus, when an edge alternates between two hues in the same location, the visual system selects the more salient hue—the one with the higher Michelson contrast. However, the afterimage is the same pink, driven by the time integral of the physical, not the perceptual, adapting hues and regardless of the surround luminance. So the process of edge biasing does not transfer to the mechanism that creates afterimages.

A patch that alternates in hue between (say) light green and dark blue on a gray surround at 5–8 Hz does not simply appear to flicker. Instead, it looks predominantly light green on a dark surround, and predominantly dark blue on a light surround.

In Supplementary Movie #1, Row 1 is a control condition. The two squares alternate slowly in synchrony, both being first the same light green (#66FF33) and then the same dark blue (#000088). The two squares are visibly the same hue at any time, although the light gray background makes the left-hand square look slightly darker.

Appearances are very different Row 2 of Supplementary Movie #1, where the two squares still flicker in synchrony between the same light green and dark blue as before, but the flicker rate is speeded up to 8 Hz (16 fps). Now their surrounds make them look very different; the left-hand square on the light gray surround looks predominantly dark blue, while the right-hand square on the dark gray surround looks predominantly light green. This is because on the light gray surround on the left, the dark blue contrasts strongly but the light green is almost equiluminous, so the more contrasty blue predominates. Conversely, on the dark gray surround on the right, the light green contrasts strongly but the dark blue is almost equiluminous, so the more contrasty green predominates. Thus, in Row 2, the visual system preferentially sees the frame that has the higher luminance contrast, blueish on the left and greenish on the right, and largely ignores the low-contrast frame with which it alternates over time. In general, when a high-contrast and a low-contrast edge of different colors alternate in the same location, the visual system is able to accept the high-contrast edge and virtually ignore the low-contrast edge. We have called this effect “flicker-augmented contrast” (FAC; Anstis & Ho, 1998). This salience-based edge selection is not a form of color induction. The surrounds are achromatic so they cannot induce any colors. Instead, they *select* colors from among the two (green and blue) that are already available in the square, always favoring the high-contrast hue. In short, the two squares contain identical flickering colors, yet they look very different. There may be an underlying process that computes hue saliences based upon edge contrasts. FAC tends to fall off at flicker rates >8 Hz, suggesting somewhat sluggish computations. In short, Row #1 in Supplementary Movie #1 gives a slowed-down version of Row #2 with the same alternations of the same colors left and right to show what is happening, and in Row #3, the two hollow squares are filled with a static mixture of the alternating light green and dark blue colors, which gives a dark turquoise. Note that the light and dark backgrounds do not alter the perceived hues in Rows #1 and #3 (apart from small lightness changes). But in Row #2, FAC makes the two squares look different in hue, blueish on the left and greenish on the right.

What color are the negative afterimages from these hollow squares? Loomis (1978) showed that both steady light and flickering light can produce afterimages. Would the afterimages also undergo FAC like the adapting stimuli and be disproportionately weighted by the higher contrast color in the stimulus? In this case, they would look different in the left and right squares, being complementary to the *perceptually* different adapting colors. Or would the afterimages act like a time exposure in a camera and respond merely to the summed hue and intensity at each point over time? In this case, they would be driven by the *physically* identical stimuli and have the same subjective hue in all the squares. Stated differently, if the afterimages in the squares are complementary to light green and dark blue, they must be built up *after* contrast edges have been compared and hue-saliences computed; but if they are simply complementary to turquoise (i.e., pink) in both squares of Row #2, then they must arise from an early, dumb, passive process analogous to a time exposure in a camera and *preceding* any image analysis.

The flickering adaptors in Supplementary Movie #1 alternate every few seconds with a white test field containing thin black lines that outline the negative afterimages. It has been shown that such outlines make afterimages much easier to see (Daw, 1962; Van Lier, Vergeer, & Anstis, 2009). Please inspect Supplementary Movie #1, letting it loop several times to build up an afterimage. If FAC applied to negative afterimages, then the afterimages from the perceptually dark blue and light green adapting squares would be, respectively, light yellow and dark magenta. But we never found such afterimages. Instead, all six adapting squares—alternating at ∼1 Hz in Row 1, flickering at 8 Hz in Row 2, and static in Row 3—gave negative afterimages of an identical pink hue (yellow + magenta = pink). The afterimages are slightly darker in the right half, but all afterimages have the same pink hue.

These results apply to other color pairs, if one color is light and the other is dark. Supplementary Movie #2 shows similar results with dark red (#880000) + light yellow (#FFFF66), in which all six squares give negative afterimages of an identical blue hue. We conclude that the visual system generates afterimages *before* biasing the edges that lead to FAC.

We conclude that FAC can change the apparent color of flickering stimuli on different surrounds but cannot change afterimages, which are driven by the time integral of the physical, not perceptual, adapting colors.

## Supplementary Material

Supplementary material

Supplementary material

## Supplemental Material

The online supplements [appendices/data supplements/etc.] are available at http://journals.sagepub.com/doi/suppl/10.1177/2041669517699414.
